# Investigating Origins of Ventricular Arrhythmia Arising From Right Ventricular Outflow Tract and Comparing Initial Ablation Strategies

**DOI:** 10.3389/fcvm.2021.727546

**Published:** 2021-10-08

**Authors:** Zhi Jiang, Qifang Liu, Ye Tian, Yidong Zhao, Wei Liu, Longhai Tian, Jing Huang, Shui Tian, Yaxi Zheng, Long Yang

**Affiliations:** ^1^Cardiology Department, Guizhou Provincial People's Hospital, Guiyang, China; ^2^Guizhou Provincial Cardiovascular Disease Institute, Guiyang, China; ^3^Guizhou Medical University, Guiyang, China

**Keywords:** pulmonary valve, pulmonary sinus cusp, premature ventricular contraction, radiofrequency, activation mapping

## Abstract

**Background:** The origin distribution in right ventricular outflow tract (RVOT) ventricular arrhythmias (VAs), as well as the initial ablation effectiveness of reversed U-curve method and antegrade method, remains unclear.

**Objectives:** To investigate the origin distribution of RVOT-type VAs and compare the initial ablation effectiveness of the two methods.

**Method:** Consecutive patients who had idiopathic RVOT-type VAs were prospectively enrolled. After activation mapping, patients were randomly assigned to supravalvular strategy using the reversed U-curve or subvalvular strategy using the antegrade method. The primary outcome was initial ablation (IA) success, defined as the successful ablation within the first three attempts.

**Results:** Sixty-one patients were enrolled from November 2018 to June 2020. Activation mapping revealed that 34/61 (55.7%) of the earliest ventricular activating (EVA) sites were above the pulmonary valves (PVs). The IA success rate was 25/33 (75.8%) in the patients assigned to supravalvular strategy as compared with 16/28 (57.1%) in those assigned to subvalvular strategy (*p* = 0.172). Multivariate analysis revealed a substantial and qualitative interaction between the EVA sites and IA strategies (*p*_interaction_ < 0.001). Either strategy had a remarkably higher IA success rate in treating its ipsilateral EVA sites than contralateral ones (*p* < 0.0083).

**Conclusion:** Of the idiopathic RVOT-type VA origins, half were located above the PV. The supravalvular and subvalvular strategies did not differ in IA success rates. However, they were complementary to reveal the EVA sites and facilitate ipsilateral ablation, which produces a significantly higher IA success rate.

**Clinical Trial Registration:** Chinese Clinical Trial Registry number, https://www.chictr.org.cn/showproj.aspx?proj=45623, ChiCTR2000029331.

## Introduction

Ventricular arrhythmias (VAs) are one of the most common arrhythmias encountered in clinical practice. VAs with left bundle branch block and inferior axis morphology frequently originate from right ventricular outflow tract (RVOT) (1). Catheter ablation of RVOT-type VAs by the antegrade method has been well established (2). The antegrade method is based on the general concept that the VA origins are mainly beneath the pulmonary valve (PV) (3). However, myocardial extensions above the PV are common (4). VAs originating from the pulmonary sinus cusps (PSCs) and pulmonary artery (PA) consist 26.6–90% of the RVOT-type VA cases in retrospective studies (5–7). A recent study reported that the reversed U-curve method had a 90% success rate in unselected patients with RVOT-type VAs (6). Subsequent studies found that the reversed U-curve method trended a higher immediate and long-term success as compared with the antegrade method (6, 8, 9).

Whether the ECG pattern is significantly different between VA origins above and beneath PVs is still unknown (8–10). The widely accepted stepwise strategy is when the antegrade method fails, then consider supravalvular origins (11). However, targeting the earliest ventricular activation (EVA) site is a key predictor for successful ablation of focal originating VA (12). One common cause of ablation failure is that the target is not close enough to form a lesion that covers the origin. The stepwise strategy initiated by the antegrade method may target the “exits” and underestimated supravalvular origins because the interposed PVs would hinder catheter contact and blur electric signals for activation mapping (7). The recent study using a supravalvular strategy initiated with reversed U-curve method reported a subset of VAs originating from the PSC junction that was refractory to the reversed U-curve method (13). When mapping by antegrade method, the earliest activation time beneath the PV was 13.2 ± 4.2 ms earlier, indicating subvalvular origins. These studies not only questioned the origin distribution in RVOT-type VAs but also questioned the ablation effectiveness of both the antegrade and reversed U-curve method.

We hypothesized that idiopathic RVOT-type VAs consisted of a considerable proportion of supravalvular origins. The ablation effectiveness of reversed U-curve and antegrade method is different according to the EVA sites. In this prospective single-center open-label randomized controlled trial, we aimed to investigate the distribution of EVA sites in patients with idiopathic RVOT-type VAs and compare the initial ablation success rate of the reversed U-curve method and the antegrade method. A secondary analysis is prespecified to measure the interaction of the EVA site and method.

## Method

### Study Population

Symptomatic patients with monomorphic RVOT-type VAs and refractory to at least one antiarrhythmic agent were eligible. Patients were excluded if they had structural heart disease or successful ablation outside the RVOT. Written informed consents were provided before the procedures. The study was approved by the ethics committee of Guizhou Provincial People's Hospital.

### Study Design

**Figure 1:**
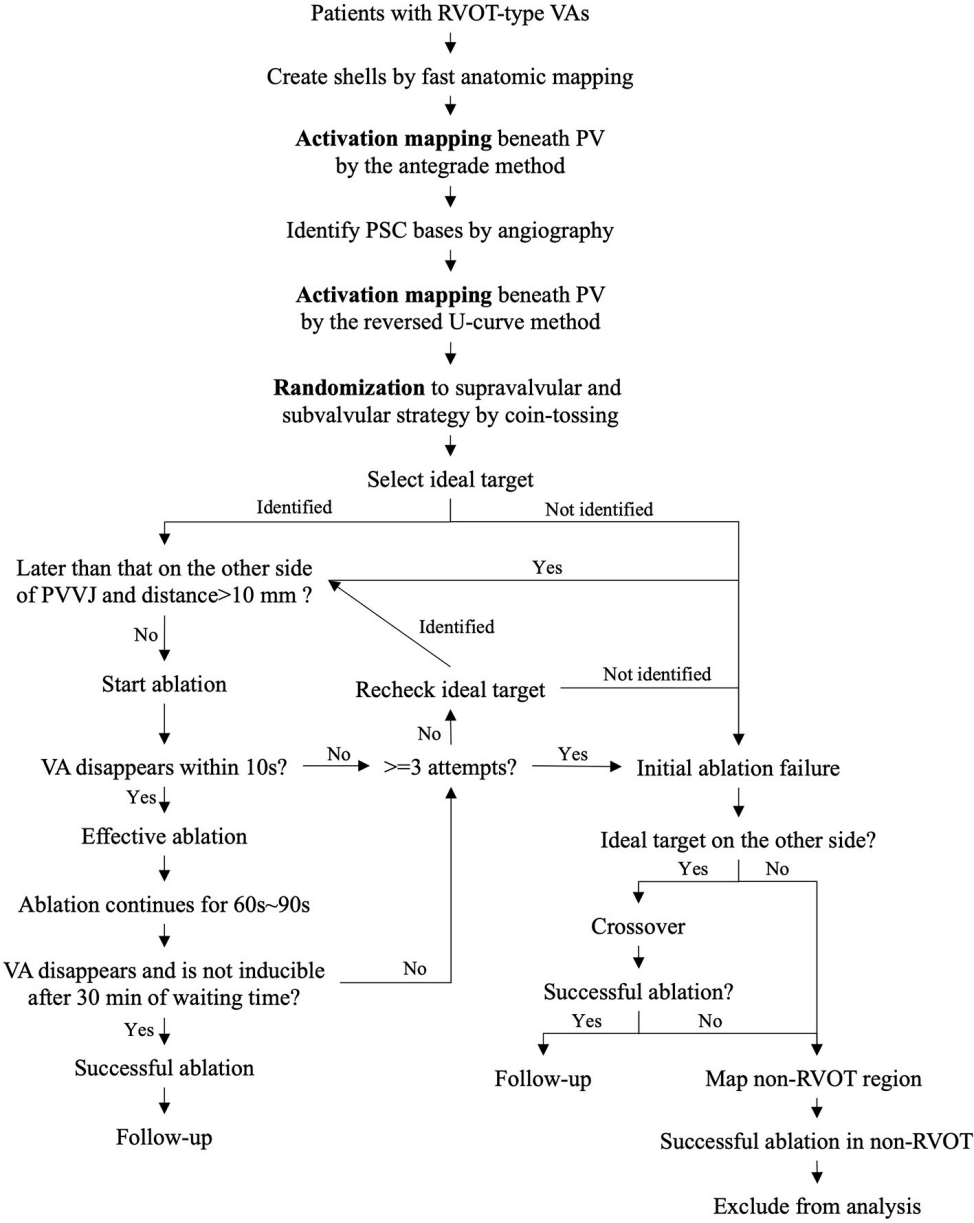
Study design. PVVJ, pulmonary valve–ventricle junction; RVOT, right ventricular outflow tract; VA, ventricular arrhythmia.

The present study is a prospective single-center open-label randomized controlled trial. The study design is illustrated in Figure 1. After activation mapping of RVOT, PSCs, and PA, the patients were randomly assigned to initial ablation (IA) with the supravalvular strategy using the reversed U-curve method and the subvalvular strategy using the antegrade method. Randomization was performed by tossing a coin. Ablation attempts were performed with a power of 30 W, a duration of 10 s, and an irrigation rate of 17 ml/min. Effective ablation was defined as suppression of VA by the ablation attempts. Isoproterenol was administrated to increase the frequency of premature ventricular contraction if necessary. Once effective ablation was identified, ablation was continued for 60–90 s. Each ablation application, including ablation attempts, were counted. Successful ablation was defined as non-inducibility of the clinical VAs with isoproterenol elicitation for 30 min. IA success was defined as the successful ablation within the first three ablation applications by the assigned strategy. IA failure included the following situations. Firstly, successful ablation was not achieved with more than three ablation attempts by the assigned strategy. Secondly, the IA targets and EVA sites were contralateral to the PVs, and the distance from the EVA site to the pulmonary valve–ventricle junction (PVVJ) was more than 10 mm. Thirdly, the operator had the discretion to declare IA failure after at least one ablation application for the patients whose targets and EVA sites were contralateral to the PVs. Patients with IA failure were managed with the other ablation strategy.

### Study Procedure

**Figure 2:**
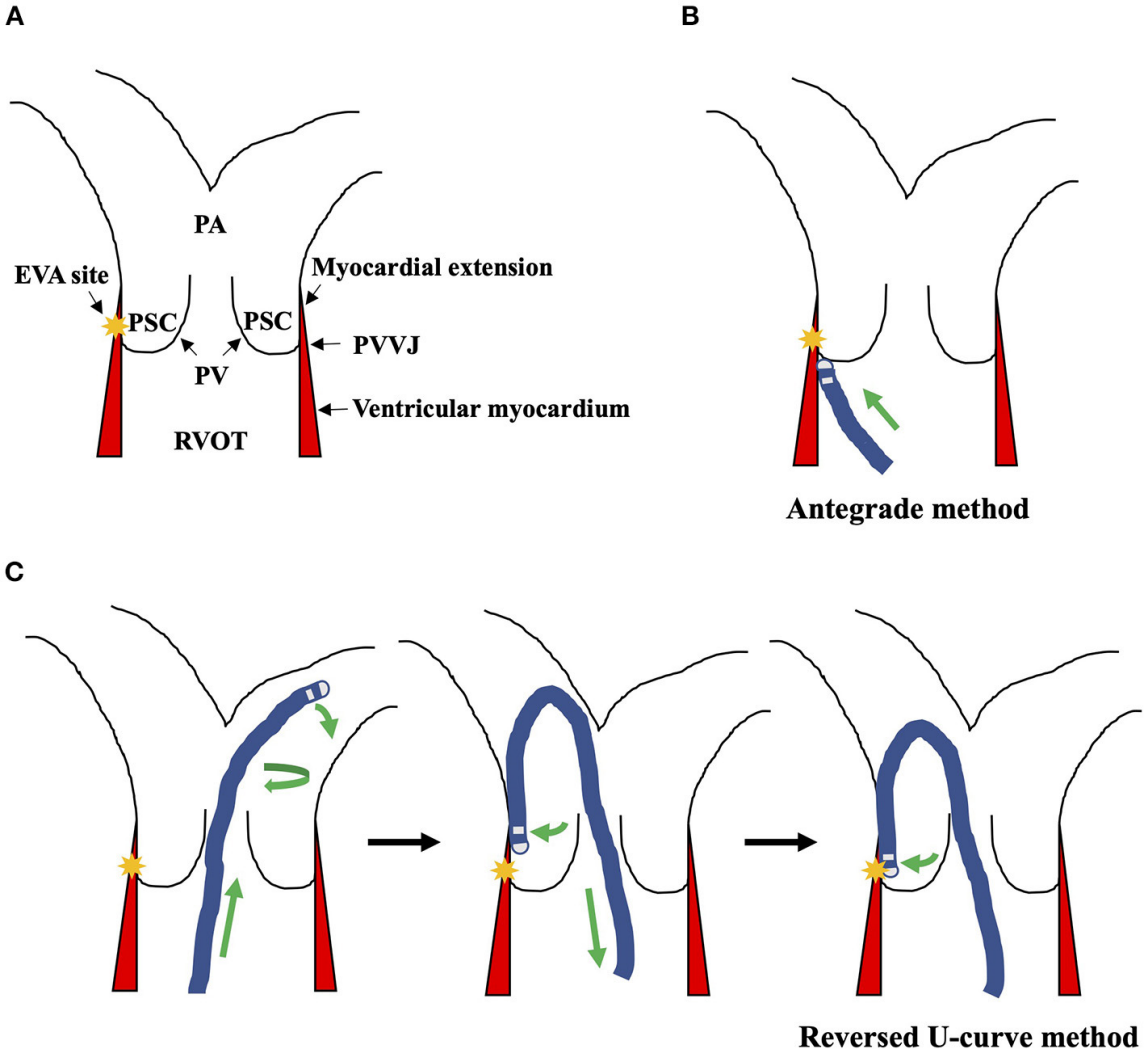
Illustration of the anatomy and the ablation strategies. **(A)** The PVVJ was where the PVs adhere to the ventricle wall. The myocardial extensions were the ventricular musculatures that extended above the PVVJ. The yellow star indicated the EVA site. **(B)** Targeting the EVA site with the antegrade method. **(C)** The reversed U-curve was created in the bifurcation of PA by curving, advancing, and torquing the ablation catheter. Then, the reversed U-curve was loosened to contact the PA wall and retracted into PSC to engage the EVA site. EVA, earliest ventricular activating; PA, pulmonary artery; PSC, pulmonary sinus cusp; PV, pulmonary valve; PVVJ, pulmonary valve–ventricle junction; RVOT, right ventricular outflow tract; VA, ventricular arrhythmia.

Antiarrhythmic medications were stopped for at least five half-lives before the procedure. Isoproterenol 1–5 μg/min was administered intravenously to provoke the clinical VAs if necessary. An 8F saline irrigating catheter (Thermocool, Biosense Webster, Diamond Bar, CA, USA) was advanced to the RVOT region under fluoroscopy through the femoral vein. A long sheath (SL1, Swartz or Preface) was used to facilitate catheter stability. For mapping of the PSCs, the reversed U-curve of the catheter was created in the bifurcation of PA, then pulled back into the PSCs, and confirmed by angiography by injecting contrast through the irrigating tube of the catheter (Figure 2) (2). PVVJ was determined by the resistance while retracting the catheter. Before ablation, the angiography was performed again to confirm the catheter's location.

Activation mapping was completed by a three-dimensional mapping system (CARTO 3, Biosense Webster, Diamond Bar, CA, USA). Bipolar and unipolar electrograms were filtered at 30 to 500 Hz and 0.05 to 400 Hz. Local activating time (LAT) was determined by the correlation of earliest rapid downstroke of unipolar signal with the first sharp peak of the bipolar electrogram. The EVA site had the longest time interval from LAT to ECG reference. The distance from the EVA site to PVVJ was measured on CARTO3. IA targets were selected from the EVA sites with the steepest Q waves in the unipolar and bipolar electrogram (14). If the assigned strategy could not reach the IA targets, the less earlier but closest sites to the IA targets along the PVVJ were targeted.

### Study Outcome

The primary outcome was IA success and ablation applications. Secondary outcomes included immediate success, periprocedural complications, and VA recurrence. Clinical PVC frequency more than 10% of baseline or any episode of VT documented by 24-h Holter was termed VA recurrence (15). An independent events committee judged all adverse events and their relation to ablation.

### Follow-Up

All patients were followed up in the cardiology clinic at 1, 3, 6, and 12 months after the procedure. Twenty-four-hour Holter monitoring was scheduled on each follow-up visit.

### Statistical Analysis

The sample size was estimated by the time to VA elimination since ablation and ablation applications (7, 9, 16). The average IA successful rate (ϕ) was assumed 85% and the difference (δ) was assumed 20%. For a power of 90% (*Z*_p_ = 1.28) at a two-sided alpha level of 0.05 (*Z*_α_ = 1.96), 134 patients were needed. Assuming a dropout rate of 20%, 160 patients were planned to be enrolled. Due to the significant interaction between the EVA sites and IA strategy, after reviewing the data, the ethics committee and the leadership discontinued the trial in July 2020.

The primary and secondary outcomes were analyzed using the intention-to-treat (ITT) principle. The logistic regression model using frontward selection (Wald) was applied to test the multiplicative interaction between the EVA sites and strategies. Their addictive interaction of absolute excess risk due to interaction (AERI) was calculated (17). Continuous variables were expressed as mean ± standard deviation or median (interquartile range). Categorical variables were expressed as number (frequency %). For comparison of patient characteristics, differences of continuous variables were tested using Student's *t*-test if normally distributed; otherwise, Mann–Whitney *U* test was used. Categorical variables were compared using the chi-square test if the expectant frequency is more than 5; otherwise, Fisher exact test was used. The VA recurrence was compared based on a time-to-first-event analysis using the log-rank test. A two-tailed *p*-value of less than 0.05 was considered statistically significant. For multiple comparisons of six contrasts among subgroups based on the EVA sites and IA strategies, a two-tailed *p*-value of less than 0.0083 was considered statistically significant using Bonferroni correction. All statistical analysis was performed using SPSS 26.

## Results

### Patient Population and Characteristics

**Figure 3:**
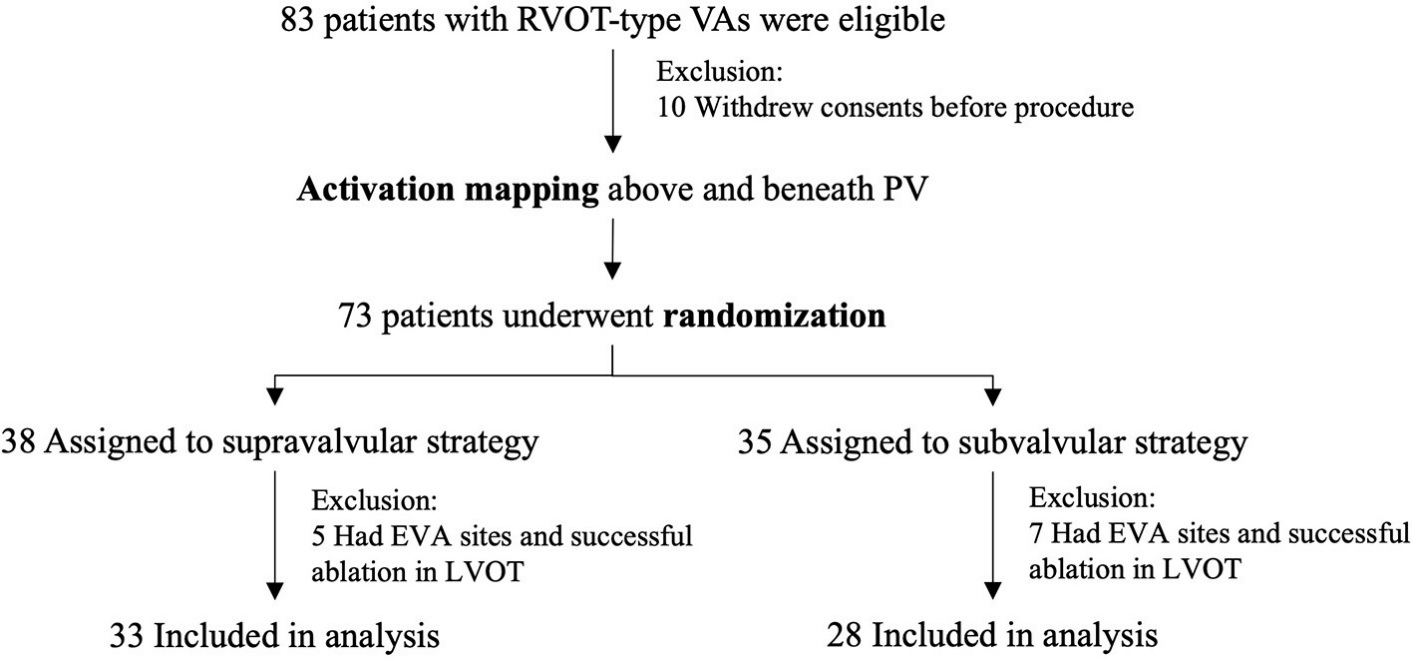
Patient flow and randomization. EVA, earliest ventricular activating; PV, pulmonary valve; PVC, premature ventricular contraction; RVOT, right ventricular outflow tract; VA, ventricular arrhythmia.

**Figure 4:**
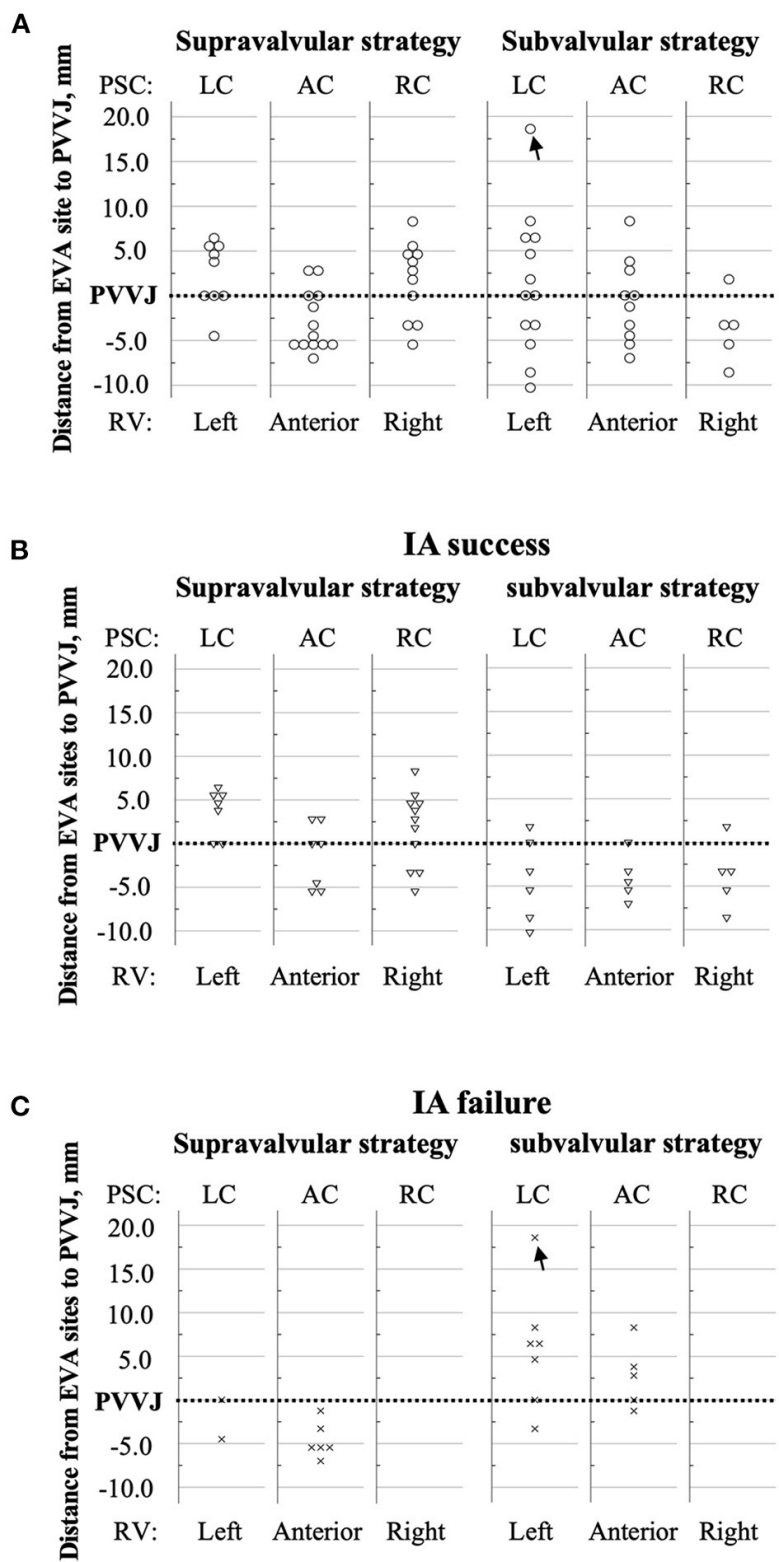
Distances from the EVA sites to the PVVJ. The RVOT was arbitrarily divided by STJ, PVVJ, and longitudinal extensions of PSC commissures. The dotted line denotes PVVJ. The markers that coincided with the PVVJ located at the base of PSCs. The *y*-axis represents the distance from each EVA site to PVVJ. **(A)** Circles denote the EVA sites in total patients. **(B)** The triangles denote the EVA sites in the patients who had IA success. **(C)** The crosses denote the EVA sites in the patients who had IA failure. The black arrow marks the EVA site in PA. AC, anterior cusp; EVA, earliest ventricular activating; LC, left cusp; PA, pulmonary artery; PSC, pulmonary sinus cusp; PVVJ, pulmonary valve–ventricle junction; RC, right cusp; RV, right ventricle; RVOT, right ventricular outflow tract; STJ, sinotubular junction.

Eighty-three patients were eligible between November 2018 and June 2020 (Figure 3). Ten patients withdrew consents, and 12 had successful ablation in the left ventricular outflow tract. Thirty-three patients assigned to supravalvular strategy and 28 assigned to subvalvular strategy were included in the analysis. Baseline characteristics between the two groups were similar (Table 1); 34/61 (55.7%) of the EVA sites were above PV, and 33/34 (97.1%) were within 10 mm above PVVJ (Figure 4A). The overall EVA site distribution was not significantly different (*p* = 0.447).

**Table 1:** Patient characteristics at baseline.

**Characteristic**	**Supravalvular strategy**	**Subvalvular strategy**	***P* value**
	**(*N* = 33)**	**(*N* = 28)**	
Age, yr	41.9 ± 16.1	43.2 ± 13.2	0.729
Female	25 (75.8)	22 (78.6)	1.000
Duration of symptoms, month	24.0 (2.0, 60)	9.0 (2.5, 36)	0.340
PVC frequency per 24 h	21,454 (15,004, 27,138)	23,138 (15,969, 28,321)	0.252
Ventricular tachycardia	8 (24.2)	4 (14.3)	0.519
**EVA sites**			
Above PV	20 (60.6)	14 (50.0)	0.447
Beneath PV	13 (39.4)	14 (50.0)	
Distance from EVA site to PVVJ, mm	0.0 (−4.6, 4.3)	−0.7 (−5.0, 3.7)	0.593
*Difference of EVAs, ms	4.0 (−4.5, 6.5)	0.5 (−4.8, 5.0)	0.353
**Locations**			
Pulmonary artery	0 (0.0)	1 (3.6)	
Left cusp	8 (24.2)	7 (25.0)	
Anterior cusp	4 (12.1)	5 (17.8)	
Right cusp	8 (24.2)	1 (3.6)	
Left RVOT	1 (3.0)	5 (17.8)	
Anterior RVOT	9 (27.3)	5 (17.8)	
Right RVOT	3 (9.1)	4 (14.3)	
**Endocardial electrogram of EVA sites**			
Bipolar QR morphology	29 (87.9)	25 (88.9)	0.976
Unipolar QS morphology	33 (100)	28 (100)	NA
Sharp potential reversal			
Above PV	21 (63.6)	16 (57.1)	0.793
Beneath PV	5 (15.2)	3 (10.7)	0.715
**Echocardiography**			
Right ventricular diameter, mm	16.0 (15.0, 17.4)	17.0 (15.0, 18.0)	0.252
RVOT diameter, mm	25.0 (22.0, 25.0)	23.0 (22.0, 25.0)	0.079
Pulmonary artery diameter, mm	18.0 (16.3, 19.0)	18.0 (17.0 19.0)	0.993
Left ventricular, mm	44.5 ± 3.6	44.1 ± 3.4	0.662
Ejection fraction, %	61.7 ± 4.1	62.9 ± 4.3	0.260

Continuous values are mean ± SD or quantile. Categorical values are n (%). *Difference of EVAs was calculated by EVA_abovePV_ minus EVA_beneathPV_. EVA, earliest ventricular activating; PV, pulmonary valve; PVC, premature ventricular contraction; PVVJ, pulmonary valve-ventricle junction; RVOT, right ventricular outflow tract.

### Primary Outcomes

IA success rate was 25/33 (75.8%) by supravalvular strategy and 16/28 (57.1%) by subvalvular strategy (*p* = 0.172) (Table 2). Supravalvular strategy trended fewer ablation applications as compared to the subvalvular strategy [1.0 (1.0, 3.5) vs. 2.0 (2.0, 4.0), *p* = 0.060] (Table 3). The operators declared IA failure in two patients assigned to subvalvular strategy. One had the EVA site 18.6 mm above PVVJ in the PA without IA application. The other had the EVA site 6.5 mm above PVVJ with two unsuccessful IA applications. The EVA sites with IA success or failure showed an ipsilateral or contralateral propensity to IA strategy (Figures 4B,C).

**Table 2:** IA success.

	**Total patients** ***N*** **=** **61**			
	**Supravalvular strategy *N* = 33**	**Subvalvular strategy *N* = 28**	**^**a**^*P* value**	**HR (95 CI; *P* value)**
All	25/33 (75.8)	16/28 (57.1)	0.172	
EVA site				*P*_interaction_ < 0.001
Supravalvular	19/20 (95)	4/14 (28.6)	<0.001	HR 0.021 (0.002–0.214; 0.001)
Subvalvular	6/13 (46.2)	12/14 (85.7)	0.046	HR 7.0 (1.098–44.607; 0.039)
^b^*P* value	0.003	0.006		

*Values are n (%). ^a^Supravalvular strategy vs. subvalvular strategy. ^b^Supravalvular EVA site vs. subvalvular EVA site. CI, confidence interval; EVA, earliest ventricular activating; IA, initial ablation; HR, hazard ratio*.

**Table 3:** Ablation applications.

		**Supravalvular strategy *N* = 33**	**Subvalvular strategy *N* = 28**	**^**a**^*P* value**
**All**				
Ablation applications	1	18/33 (54.5)	6/28 (21.4)	0.060
	2	4/33 (12.1)	9/28 (32.1)	
	3	3/33 (9.1)	3/28 (10.7)	
	4	5/33 (15.2)	8/28 (28.6)	
	5	2/33 (6.1)	2/28 (7.1)	
	6	1/33 (3.0)	0/28 (0.0)	
**EVA site**				
Supravalvular	*N* = 20	*N* = 14		
Ablation applications	1	15/20 (75.0)	0/14 (0.0)	0.001
	2	3/20 (15.0)	5/14 (35.7)	
	3	1/20 (5.0)	1/14 (7.1)	
	4	0/20 (0.0)	7/14 (50.0)	
	5	1/20 (5.0)	1/14 (7.1)	
	6	0/20 (0.0)	0/14 (0.0)	
Subvalvular	*N* = 13	*N* = 14		
Ablation applications	1	3/13 (23.1)	6/14 (42.9)	0.035
	2	1/13 (7.7)	4/14 (28.6)	
	3	2/13 (15.4)	2/14 (14.3)	
	4	5/13 (38.5)	1/14 (7.1)	
	5	1/13 (7.7)	1/14 (7.1)	
	6	1/13 (7.7)	0/14 (0.0)	
^b^*P* value		0.003	0.058	

*Values are n (%). ^a^Supravalvular strategy vs. subvalvular strategy. ^b^Supravalvular EVA site vs. subvalvular EVA site. EVA, earliest ventricular activating*.

### Secondary Outcomes

**Figure 5:**
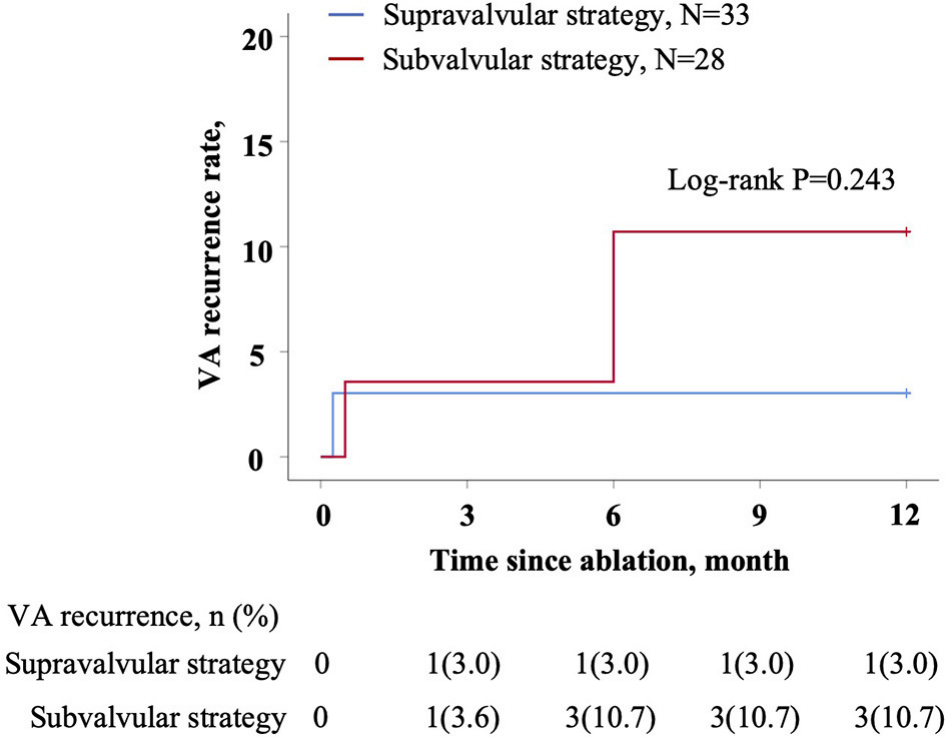
Kaplan–Meier estimates of the accumulative VA recurrence of 12 months. Patient number (frequency) was shown at the bottom. *p*-value was shown on the graph. IA, initial ablation; VA, ventricular arrhythmia.

The 20 patients with IA failure all had successful ablation by the other strategy. One patient assigned to supravalvular strategy suffered from tamponade after the procedure, but recovered by urgent drainage without blood transfusion. No complications occurred in the other patients. Until June 2021, all patients reached 12 months of follow-up (Figure 5). One patient from supravalvular strategy and three from subvalvular strategy had VA recurrence (*p* = 0.243). One patient from supravalvular strategy had a second procedure after 1 month. Activation mapping revealed the same EVA site in LC, and ablation using the reversed U-curve eliminated PVC within seconds. The patient was then followed for another 12 months. No VA recurrence was noted.

### Secondary Analysis

The treatment effect was distinct between the patients with supravalvular and subvalvular EVA sites. For multiple comparison, either strategy was superior in IA success rate to treat its ipsilateral EVA sites as compared with contralateral ones (*p* < 0.0083) (Table 2). In the logistic regression model, female gender, age, presence of VT, PVC frequency, sharp potential reversal at target, EVA site (supravalvular or subvalvular), IA strategy, RVOT diameter, and PA diameter were included to adjust clinical confounders (Table 2). The EVA site was the only baseline characteristic that had a remarkable multiplicative treatment interaction (*p*_interaction_ < 0.001) with the IA strategy. The AERI was 105.2%, suggesting a large magnitude of super-addictive interaction between the EVA site and its ipsilateral strategy. In patients with supravalvular EVA sites, supravalvular strategy was associated with a lower risk of IA failure (HR 0.021, 95% CI 0.002–0.214; *p* = 0.001), whereas in patients with subvalvular EVA sites, the supravalvular strategy was associated with a higher risk of IA failure (HR 7.000, 95% CI 1.098–44.607; *p* = 0.039). The supravalvular strategy had fewer ablation applications in treating supravalvular EVA sites than subvalvular EVA sites, as well as the subvalvular strategy (*p* < 0.0083) (Table 3).

### Analysis of the Patients With Non-ipsilateral Matching

**Figure 6:**
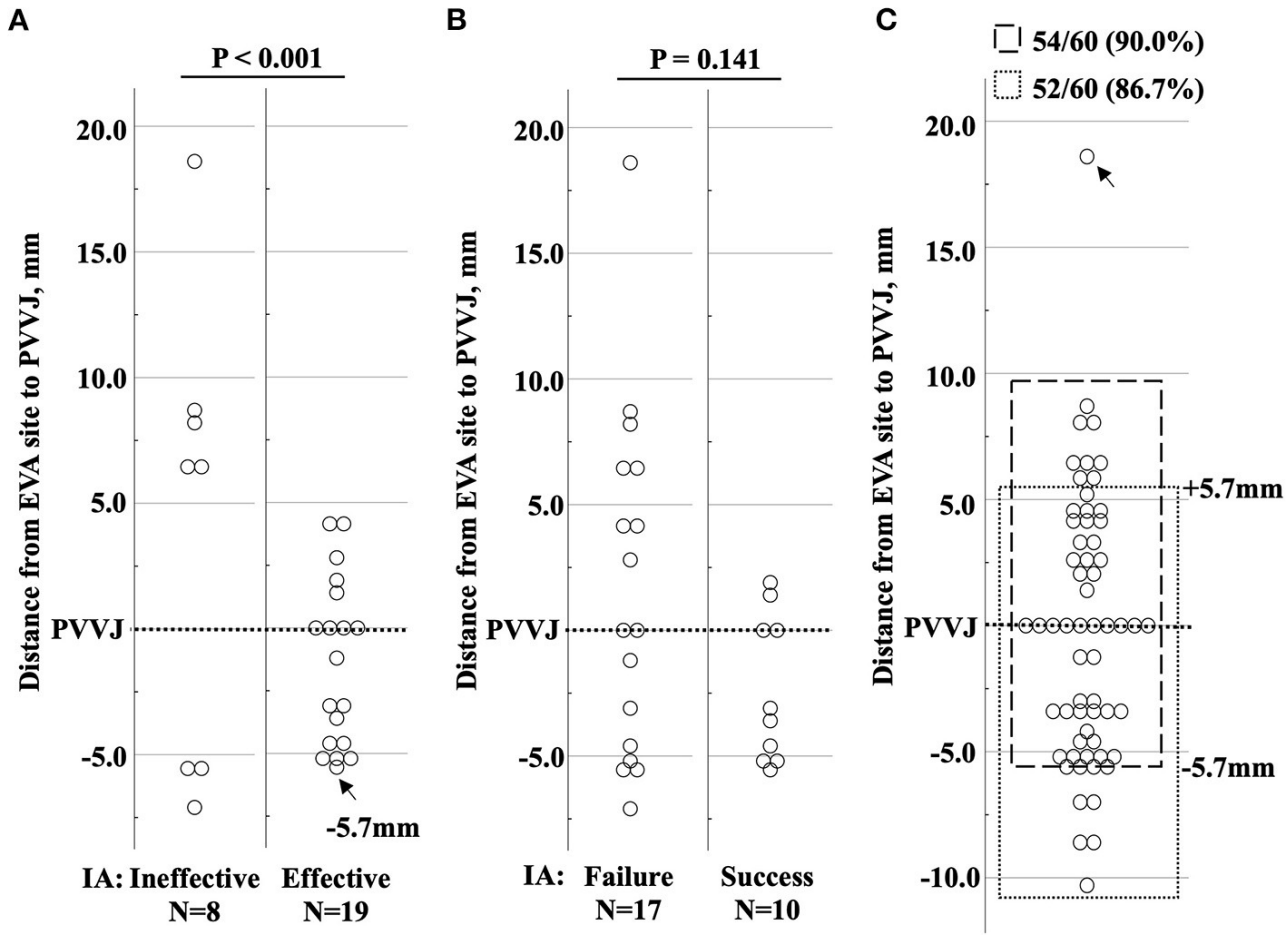
Distribution of the EVA sites in patients who had non-ipsilateral-matching IA. **(A)** The circles denote the EVA sites with ineffective and effective IA. Effective ablation was defined as suppression of VA within 10 s of radiofrequency energy delivery. The black arrow marks the farthest EVA site to PVVJ (−5.7 mm) with effective ablation. **(B)** The circles denote the EVA sites with IA success and failure. **(C)** The circles denote the EVA sites in total patients. The EVA site (black arrow) in PA was excluded. The dashed box denotes the EVA sites located from −5.7 mm to 10.0 mm, estimating that 54/60 (90.0%) patients could have effective ablation by supravalvular strategy. The dotted box denotes the EVA sites from RVOT to +5.7 mm above PVVJ, estimating that the subvalvular strategy could have effective ablation in 52/60 (86.7%) patients. EVA, earliest ventricular activating; IA, initial ablation; PA, pulmonary artery; PVVJ, pulmonary valve–ventricle junction.

Twenty-seven patients with contralateral IA underwent further analysis. IA suppressed VAs in 19/27 (70.4%) patients (Figure 6A), but only 10/27 (37.0%) had IA success (Figure 6B). A change in QRS morphology was observed in three patients assigned to the subvalvular strategy. By measuring the distances from EVA sites to PVVJ, 5.7 mm was the maximum for contralateral IA to be effective, indicating the largest heating radius of the ablation attempts. Using the distance of 5.7 mm, excluding one patient with PA origin, effective ablation was estimated 54/60 (90.0%) by the supravalvular strategy and 52/60 (86.7%) by the subvalvular strategy in the total patients (Figure 6C).

## Discussion

### Major Findings

We reported the first randomized controlled trial, which had investigated the EVA site distribution and compared the ablation effectiveness between the supravalvular strategy and the subvalvular strategy in patients with idiopathic monomorphic RVOT-type VAs. Half of the EVA sites were above the PVs, and most were within 10 mm above PVVJ. The two strategies did not differ in the IA success rates, ablation applications, complications, and long-term VA recurrence. However, the ipsilateral IA targeting the EVA sites remarkably increased the success rate and reduced ablation applications. Over 5.7 mm from the EVA sites to PVVJ was the indicator for requiring a specific method to achieve successful ablation by contralateral ablation.

### Comparison of the Strategies

Although our trial was of low power (0.345) to reveal the difference, the secondary analysis had revealed the significant and qualitative interaction between the EVA sites and IA strategies. The subgroup effect cannot be explained by chance because of its substantial magnitude, statistical significance, consistency through subgroups, and electrophysiologic rationale (18). The result reiterated the EVA site as a critical baseline characteristic for selecting the optimal method to achieve higher ablation effectiveness. The findings weakened the value of comparing the overall IA success rates between the strategies irrespective of the EVA sites. Since the contralateral IA was no longer in patients' interests and a larger sample size would not add value, the ethics committee and the leadership discontinued the study.

### Clinical Implication

By determining the PVVJ, we found that half of the RVOT-type VAs were of supravalvular origin. The result is supported by previous studies using intracardiac echocardiography (ICE) and PA angiography (6, 7). It is essential to remind the importance of PVs in catheter ablation for RVOT-type VAs. They protect supravalvular origins when using the antegrade method and prevent engaging subvalvular origins when using the reversed U-curve method. Considering the high proportion of supravalvular origins in RVOT-type VAs, activation mapping by a single method is not sufficient to reveal the true EVA sites.

We postulate that the VA suppression by the contralateral ablation was due to the increasing temperature that had temporarily suppressed the arrhythmogenicity of the VA origins but not permanently destroyed them. More ablation applications, prolonged ablation, and higher power were usually necessary to form larger lesions to achieve success (7). The “collateral damage” pattern of ablation may impair clinical outcomes and increase complication risks. Our findings suggested complete activation mapping for locating VA origins and then selecting the optimal method for ipsilateral ablation.

### Anatomical Considerations

**Figure 7:**
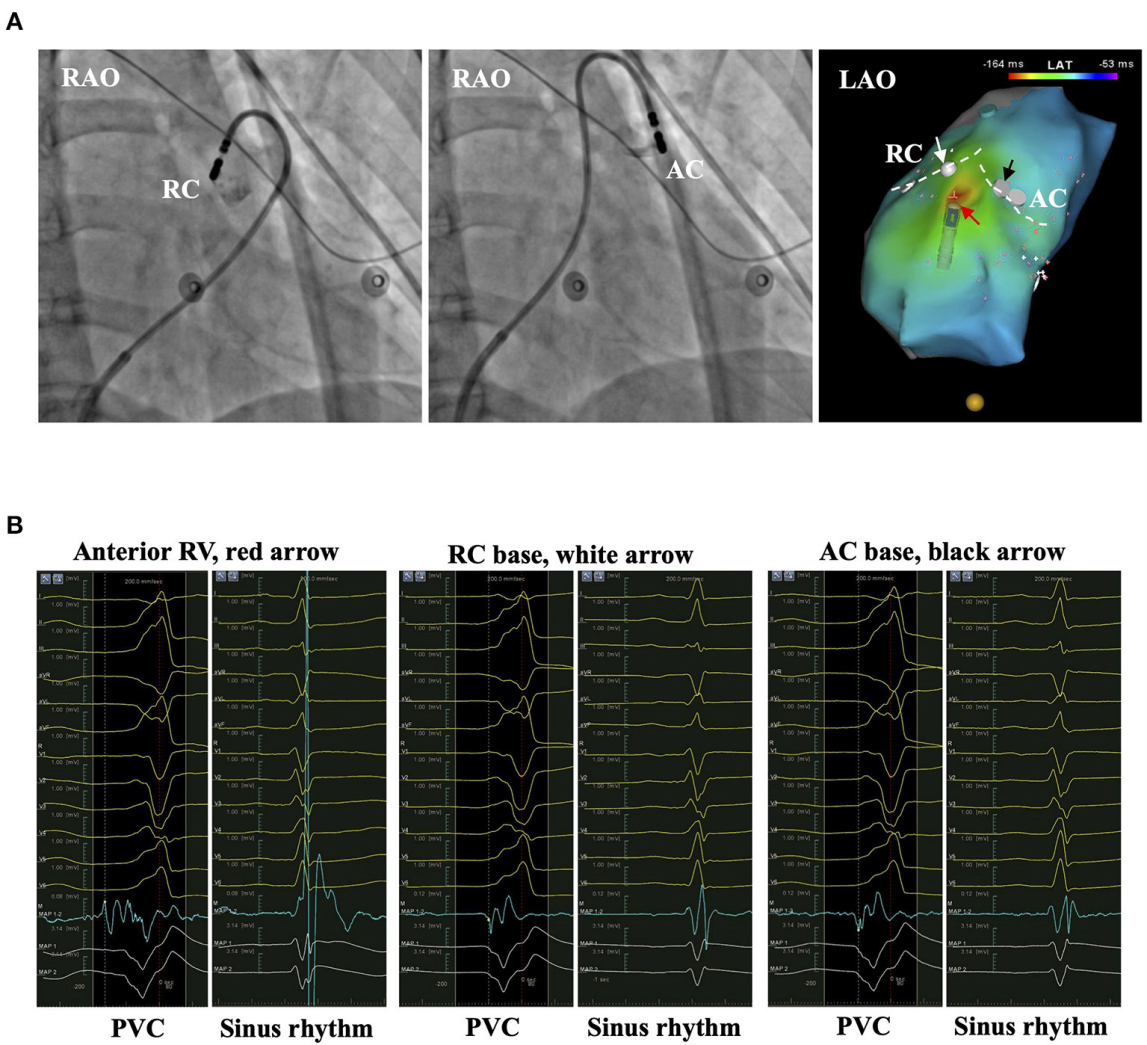
The representative patient with PVC originating from anterior RV. **(A)** The left and middle panels show the bases of RC and AC by injecting contrast from the irrigation tube of the catheter. The right panel shows the activation mapping. The white dashed line denotes the PVVJ. The red arrow marks the EVA site 8.5 mm beneath PVVJ. White and black arrows mark the less early activation site at the base of RC and AC. **(B)** The panels show the electrograms in PVC and sinus rhythm recorded at the arrow-denoted sites from left to right. The red dashed line denotes the time point of reference. The yellow dashed line denotes the LAT. AC, anterior cusp; EVA, earliest ventricular activating; LAT, local activating time; PVC, premature ventricular contraction; PVVJ, pulmonary valve–ventricle junction; RC, right cusp.

**Figure 8:**
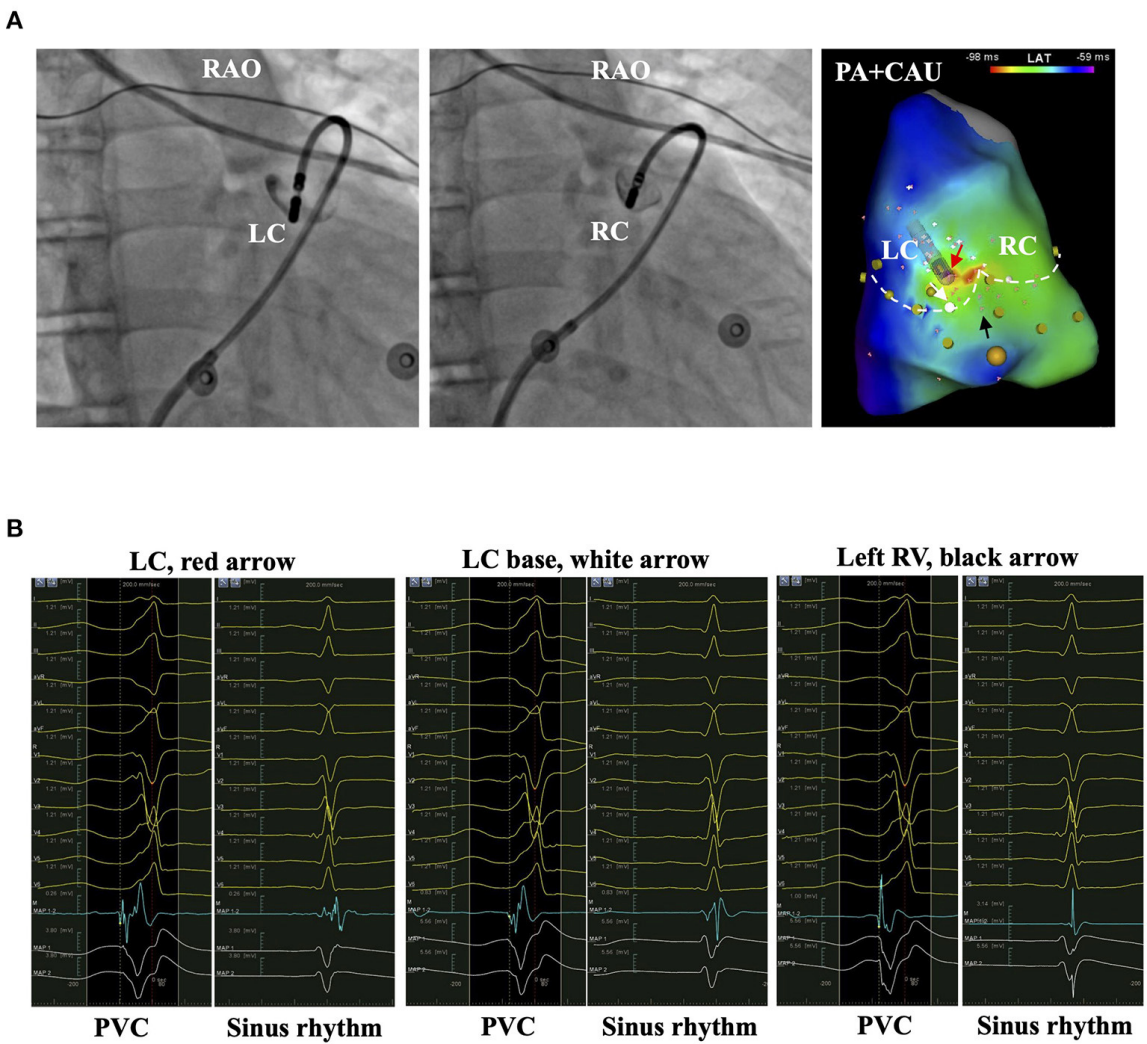
The representative patient with PVC originating from LC. **(A)** The left and middle panels show the bases of LC and RC by injecting contrast from the irrigation tube of the catheter. The right panel shows the activation mapping. The white dashed line denotes the PVVJ. The red arrow marks the EVA site 5.9 mm above PVVJ. White and black arrows mark the less early activation site at the LC base and left RV. **(B)** The panels show the electrograms in PVC and sinus rhythm recorded at the arrow-denoted sites from left to right. The red dashed line denotes the reference. The yellow dashed line denotes the LAT. EVA, earliest ventricular activating; LAT, local activating time; LC, left cusp; PVC, premature ventricular contraction; PVVJ, pulmonary valve–ventricle junction; RC, right cusp.

**Figure 9:**
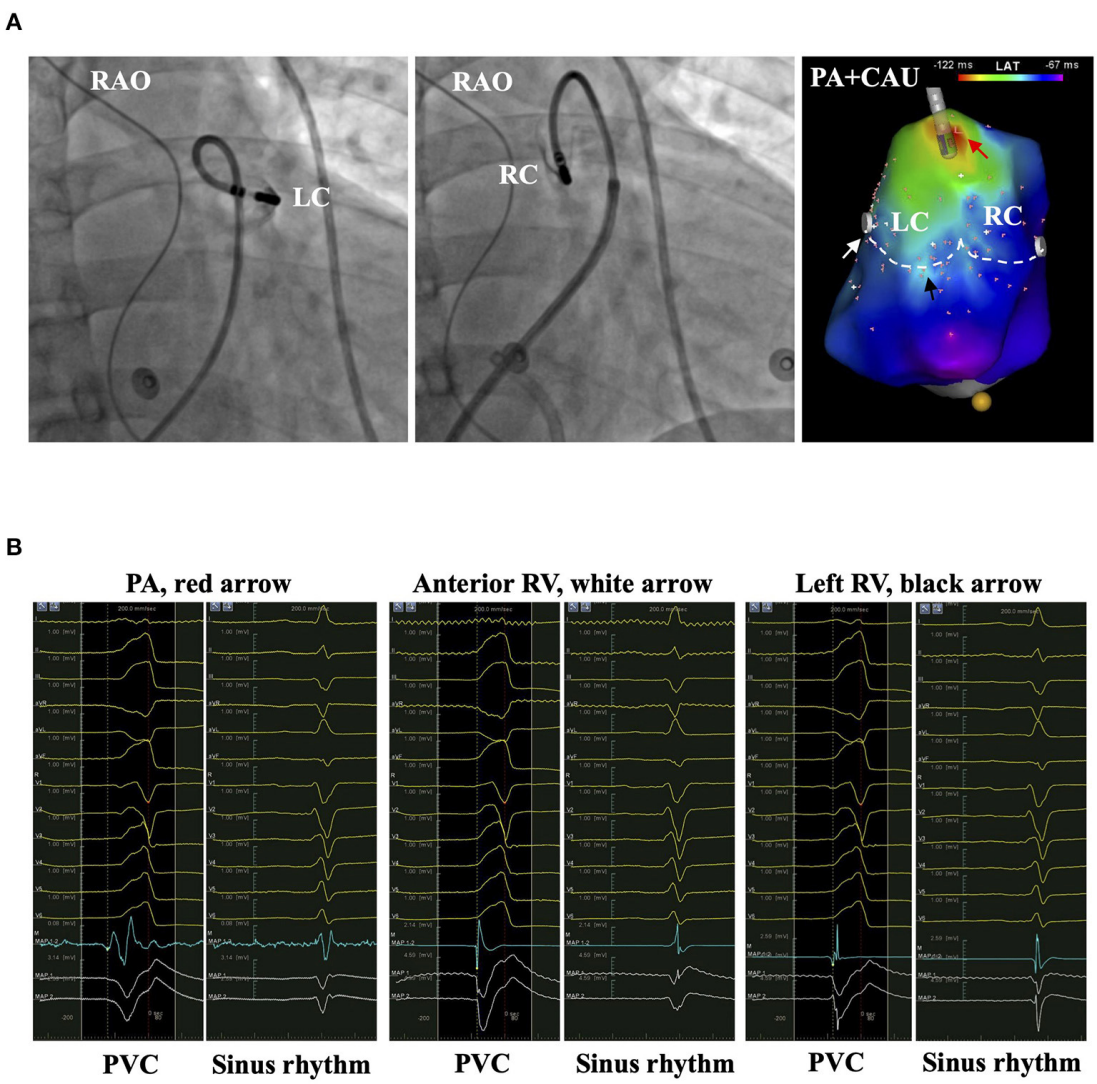
The representative patient with PVC originating from PA. **(A)** The left and middle panels show the bases of LC and RC by injecting contrast from the irrigation tube of the catheter. The right panel shows the activation mapping. The white dashed line denotes the PVVJ. The red arrow marks the EVA site 18.6 mm above PVVJ. White and black arrows mark the relative early ventricular activation site at the anterior and left RV. **(B)** The panels show the electrograms in PVC and sinus rhythm recorded at the arrow-denoted sites from left to right. The red dashed line denotes the time point of reference. The yellow dashed line denotes the LAT. EVA, earliest ventricular activating; LAT, local activating time; LC, left cusp; PVC, premature ventricular contraction; PVVJ, pulmonary valve–ventricle junction; RC, right cusp.

In the *in vitro* study using porcine myocardial slabs, 30 W and 10 s of ablation created lesions of 6.3 ± 0.4 mm in width and 2.7 ± 0.4 mm in depth (19). The lesions could cover the VA origins on the other side of PVVJ by contralateral ablation. Histological analysis of the myocardial extensions above PV found a high presence of discontinuity, myocellular hypertrophy, and fatty tissue infiltration (20). The antegrade method could have successful ablation by eliminating the single “exit” of the supravalvular origins. In our study, the effective ablation rate was estimated at 90% by either strategy in the patients with EVA sites less than 5.7 mm to PVVJ. Only 10% of the patients required a specific method. For the patient with the EVA site 8.5 mm beneath the PVVJ, the antegrade method was preferred (Figure 7). For the patient with the EVA site 5.9 mm above PVVJ, the reversed U-curve method was preferred (Figure 8). For the patient with EVA site in PA, either method would work (Figure 9). A few patients had IA failure by ipsilateral ablation because catheter stability is also a prerequisite for successful ablation. When the VA origins were close to the PVVJ, the two strategies may have equal ablation effectiveness. The strategy with better catheter stability may be preferred.

### Discordances

The overall IA success rate was lower than previous studies (6, 9). Firstly, the 30-W and 10-s ablation attempts had avoided collateral damage to VA origins from ablation at a distal target. The setting resulted in a low IA success rate in patients with contralateral ablation. Secondly, the proportion of patients with supravalvular EVA site was fewer than Zhang's cohort [(20/33 (60.6%) vs. 72/81 (88.9%)] (6). Lastly, since the antegrade method is inferior in catheter stability to the reversed U-curve method, the subvalvular strategy was more susceptible to IA failure as more ablation applications were consumed (8).

## Limitations

Our study had the following limitations. Firstly, the sample size was small. Secondly, the coin-tossing randomization did not equilibrate the EVA site distribution in groups. Thirdly, the benefits of fewer ablation applications, such as myocardial damage, total ablation time, procedural time, and X-ray exposure, were not evaluated. Fourthly, the aortic sinus of Valsalva and coronary venous system was not routinely mapped. Fifthly, the echocardiography was reassessed in 11/61 (18%) patients after the procedure. No PV dysfunction was noted. Lastly, ICE and high-density mapping were not used.

## Conclusions

In the prospective single-center open-level randomized trial, half of the origins in idiopathic RVOT-type VAs were above the PV. The supravalvular and subvalvular strategies were not different in IA success rate, ablation application, complication, and VA recurrence. However, they are complementary to reveal the true EVA sites and facilitate the ipsilateral ablation, which produces a significantly higher success rate.

## Data Availability Statement

The raw data supporting the conclusions of this article will be made available by the authors, without undue reservation.

## Ethics Statement

The studies involving human participants were reviewed and approved by the Ethics Committee of Guizhou Provincial People's Hospital. The patients/participants provided their written informed consent to participate in this study.

## Author Contributions

ZJ and QL designed the present study. ZJ, QL, YT, and LY participated in the trial as major operators. YZha, WL, LT, and JH performed the statistical analysis and verified the analytical methods. ZJ took the lead in writing the manuscript in consultation with YZhe and LY. ST aided in interpreting the results and worked on the manuscript. LY supervised the findings of this work. All authors discussed the results and contributed to the final manuscript.

## Funding

This work was supported by the Clinical Research Center Project of Department of Science and Technology of Guizhou Province [No. (2017)5405], the Guizhou Provincial High-Level Innovative Talents Project [GZSYQCC(2015)006], the Guizhou Provincial Science and Technology Foundation [No. (2019)1197], the Guizhou Provincial Science and Technology Social Development Project [No. (2018)2794], and the Youth Funds of Guizhou Provincial People's Hospital [GZSYQN(2019)19].

## Conflict of Interest

The authors declare that the research was conducted in the absence of any commercial or financial relationships that could be construed as a potential conflict of interest.

## Publisher's Note

All claims expressed in this article are solely those of the authors and do not necessarily represent those of their affiliated organizations, or those of the publisher, the editors and the reviewers. Any product that may be evaluated in this article, or claim that may be made by its manufacturer, is not guaranteed or endorsed by the publisher.

## Abbreviations

EVA: earliest ventricular activating
PA: pulmonary artery
PSC: pulmonary sinus cusp
PV: pulmonary valve
PVC: premature ventricular contraction
PVVJ: pulmonary valve–ventricle junction
RVOT: right ventricular outflow tract
VA: ventricular arrhythmia
VT: ventricular tachycardia
RF: radiofrequency.
